# Daptomycin-Impregnated PMMA Cement against Vancomycin-Resistant Germs: Dosage, Handling, Elution, Mechanical Stability, and Effectiveness

**DOI:** 10.3390/antibiotics12111567

**Published:** 2023-10-26

**Authors:** Martina Humez, Eugen Domann, Kai M. Thormann, Christian Fölsch, Rainer Strathausen, Sebastian Vogt, Volker Alt, Klaus-Dieter Kühn

**Affiliations:** 1 Institute of Hygiene and Environmental Medicine, Justus-Liebig-Universität Giessen, Schubertstrasse 81, 35392 Giessen, Germany; eugen.domann@mikrobio.med.uni-giessen.de (E.D.); kai.thormann@mikro.bio.uni-giessen.de (K.M.T.); 2 Heraeus Medical GmbH, Philipp-Reis-Str. 8-13, 61273 Wehrheim, Germany; rainer.strathausen@heraeus.com (R.S.); sebastian.vogt@heraeus.com (S.V.); 3 Institute of Microbiology and Molecular Biology, Justus-Liebig-Universität Giessen, Heinrich-Buff-Ring 26, 35392 Giessen, Germany; 4 Department of Orthopaedic Surgery, Justus-Liebig-University, Klinikstrasse 33, 35392 Giessen, Germany; christian.foelsch@ortho.med.uni-giessen.de; 5 Department of Trauma Surgery, University Medical Centre, Regensburg, Franz-Josef-Strauß-Allee 11, 93053 Regensburg, Germany; volker.alt@ukr.de; 6 Department of Orthopaedics and Trauma, Medical University of Graz, Auenbruggerplatz 5, 8036 Graz, Austria

**Keywords:** daptomycin, PMMA cement, mechanical properties, antimicrobial effectiveness, vancomycin-resistant germs, PMMA spacer

## Abstract

Background: The number of periprosthetic joint infections caused by vancomycin-resistant pathogens is increasing. Currently, no PMMA cement is commercially available to cover VRE. Daptomycin shows promising results in treating infection, offering a good safety profile and a reduced risk of developing resistance. The purpose of this in vitro study was to investigate the mechanical stability, handling properties, elution behavior, and antimicrobial effectiveness of PMMA cement loaded with three different daptomycin concentrations in comparison to commercially available antibiotic-loaded bone cement (ALBC). Methods: Mechanical properties and handling characteristics (ISO 5833, DIN 53435), HPLC elution, antimicrobial effectiveness with proliferation assay (DIN 17025), and inhibition zone testing were investigated. Results: All tested daptomycin concentrations met the ISO and DIN standards for mechanical strength. Loading of 40 g of PMMA cement with 0.5 g of daptomycin did not show any antimicrobial effectiveness, in contrast to 1.0 g and 1.5 g. PMMA cement with 1.5 g of daptomycin was the best in terms of elution and effectiveness, and it showed good ISO mechanical strength; ISO doughing was sticky for a little longer and setting was faster compared to the vancomycin-containing reference cement. Conclusion: PMMA cement containing 0.5 g of gentamicin and 1.5 g of daptomycin could be a good alternative to the already established COPAL^®^ (Wehrheim, Germany) G+V for the treatment of PJIs caused by VRE.

## 1. Introduction

Periprosthetic joint infections (PJIs) are a challenging complication in joint replacement surgery that often results in worse outcomes for the patients, especially when the causative pathogen is a multidrug-resistant germ [1] and/or the patient is at high risk [2,3]. Poly(methyl methacrylate) (PMMA) bone cements loaded with one (i.e., single-antibiotic-loaded bone cement (SALBC)) or two antibiotics (i.e., dual-antibiotic-loaded bone cement (DALBC)) are used for the prevention or treatment of PJIs, e.g., for a spacer in the interim period of a two-stage exchange procedure [4,5,6,7]. DALBC supports reducing PJIs especially well compared to SALBC [8]. Commercially available antibiotic-loaded bone cements (ALBCs) mainly contain the antibiotics gentamicin or tobramycin (aminoglycosides), vancomycin (a glycopeptide), and clindamycin (a lincosamide) [9]. The number of PJIs caused by resistant germs, including vancomycin-resistant *Staphylococcus aureus* (VRSA), vancomycin-resistant *Enterococci* (VRE), methicillin-resistant *Staphylococcus aureus* (MRSA), and methicillin-resistant *Staphylococcus epidermidis* (MRSE), is rising [10,11], and the manual addition of vancomycin to commercially available ALBCs is insufficient to cover these germs. The aforementioned bacterial species have mainly developed resistance against vancomycin and methicillin; therefore, the antibiotics used for their treatment must be adapted to the resistance pattern of the causative bacteria [12]. One antibiotic that has been found to be effective against Vancomycin-intermediate-resistant *S. aureus* (VISA), VRSA, and VRE is daptomycin; it also shows antimicrobial activity against anaerobic bacteria, but none against Gram-negative bacteria [10,13]. Daptomycin has a unique mode of action and disrupts the cell membrane integrity of bacteria [14].

There is no daptomycin ALBC commercially available, and the manual admixture of daptomycin is highly expensive (Cubicin, ~780 EUR/2 g). In clinical practice, surgeons increase the vancomycin concentration up to 4 g per 40 g of polymer powder to treat vancomycin-resistant germs. But a massively increased antibiotic concentration in PMMA spacers negatively influences their mechanical stability [15], and the risk of local and systemic kidney toxicity is highly increased. According to the “Pocket Guide to Diagnosis & Treatment of PJI” from the PRO-IMPLANT Foundation (PIF), 2 g of daptomycin can be added to a fixation cement, and 3 g of daptomycin can be added to a spacer cement [16]. The addition of more than 2 g of daptomycin results in non-ISO-compliant mechanical properties.

With this investigation, we wanted to figure out what concentration of daptomycin can be added to PMMA cement to efficiently inhibit bacterial growth and, at the same time, deliver mechanical stability according to ISO standards.

## 2. Results

### 2.1. Antimicrobial Effectiveness Determined by Proliferation Assay

**Figure 1:**
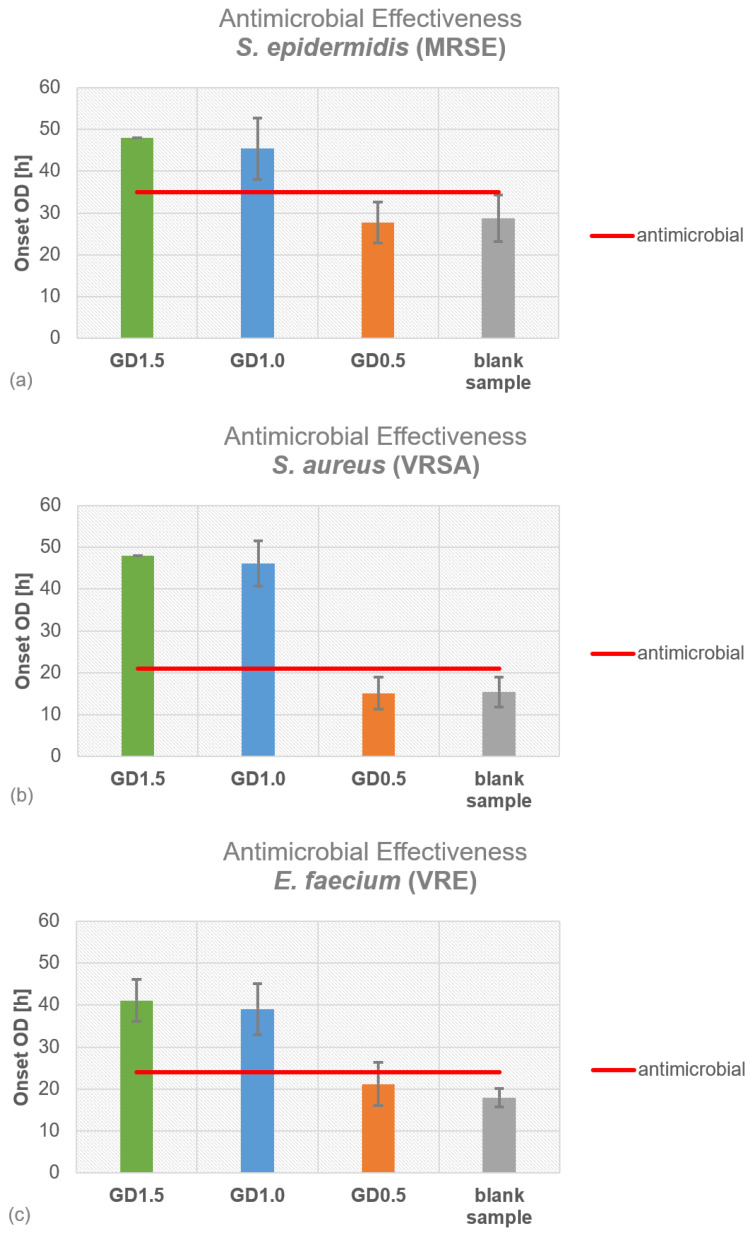

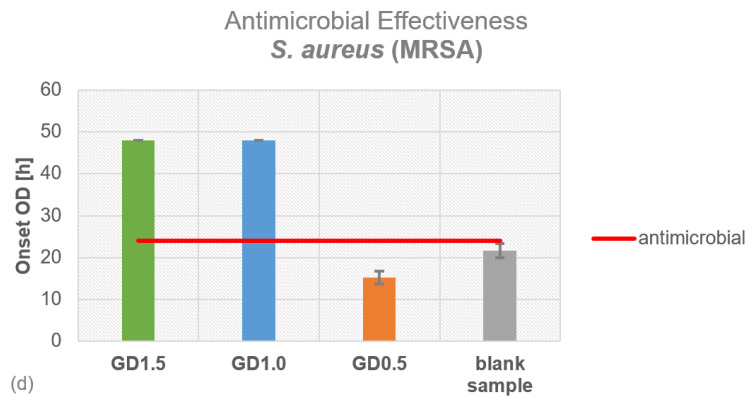
Antimicrobial effectiveness of different daptomycin concentrations (0.5 g, 1.0 g, of 1.5 g) in PMMA bone cement samples, as determined by Certika^®^ (Barranquilla, Colombia) proliferation assay for (**a**) methicillin-resistant *S. epidermidis*, (**b**) vancomycin-resistant *S. aureus*, (**c**) vancomycin-resistant *E. faecium*, and (**d**) methicillin-resistant *S. aureus*.

For methicillin-resistant *S. epidermidis* (MRSE), the blank sample and PMMA cement loaded with 0.5 g of gentamicin and daptomycin (GD0.5) did not show any antimicrobial activity (Figure 1a). GD1.0 (1.0 g of daptomycin + 0.5 g of gentamicin) and GD1.5 (1.5 g of daptomycin + 0.5 g of gentamicin) inhibited bacterial growth, and GD1.5 showed better antimicrobial properties compared to GD1.0. For vancomycin-resistant *S. aureus* (VRSA), the blank sample and GD0.5 did not show antimicrobial activity (Figure 1b), whereas GD1.0 and GD1.5 were able to inhibit bacterial growth. The same observation was made for vancomycin-resistant *E. faecium* (VRE), where only GD1.0 and GD1.5 showed antimicrobial activity (Figure 1c). For methicillin-resistant *S. aureus* (MRSA), the blank sample and GD0.5 did not inhibit bacterial growth, in contrast to GD1.0 and GD1.5. Overall, the antimicrobial efficacy of GD1.5 and GD1.0 did not differ widely, with GD1.5 showing the greatest effect on bacterial growth.

### 2.2. Antimicrobial Effectiveness Based on Inhibition Zone Tests

**Figure 2:**
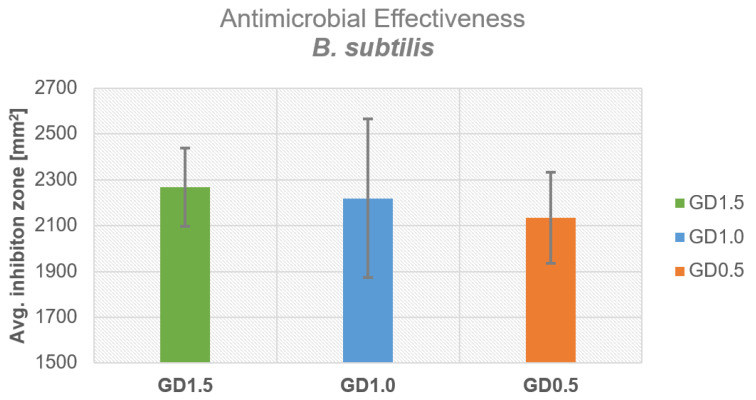
Antimicrobial effectiveness as determined by inhibition zone tests for all three different daptomycin concentrations.

The inhibition zones of daptomycin-containing PMMA cements showed the best effectiveness with a concentration of 1.5 g of daptomycin. Figure 2 shows the antimicrobial effectiveness of the tested samples relative to the amount of daptomycin added: 1.5 g led to an average inhibition zone of 2269 ± 171 mm^2^, 1.0 g led to an inhibition zone of 2219 ± 346 mm^2^, and 0.5 g led to an inhibition zone of 2134 ± 198 mm^2^. GD1.5, GD1.0, and GD0.5 showed antimicrobial effectiveness against *B. subtilis* (Figure 2).

### 2.3. Influence of the Sterilization Method

**Figure 3:**
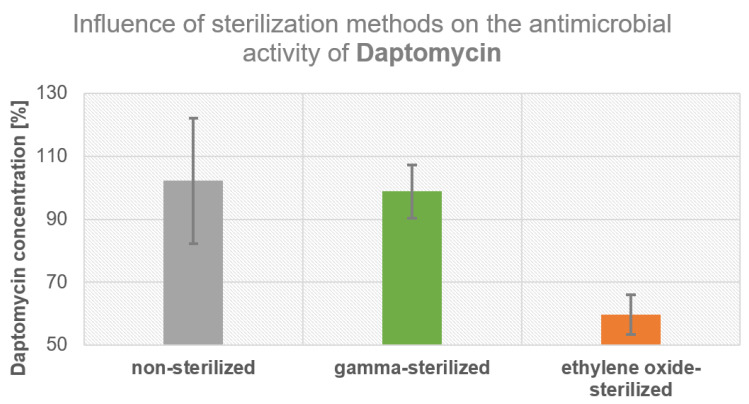
Influence of gamma and ethylene oxide sterilization on the antimicrobial effectiveness of daptomycin against *B. subtilis*, in contrast to non-sterilized daptomycin.

Non-sterilized and gamma-irradiated pure daptomycin powder showed comparable effectiveness against *B. subtilis* (Figure 3). The effectiveness of daptomycin sterilized with ethylene oxide was significantly reduced in comparison to the unsterile and gamma-irradiated powders.

### 2.4. Antibiotic Release Profile of Gentamicin, Daptomycin, and Vancomycin

**Figure 4:**
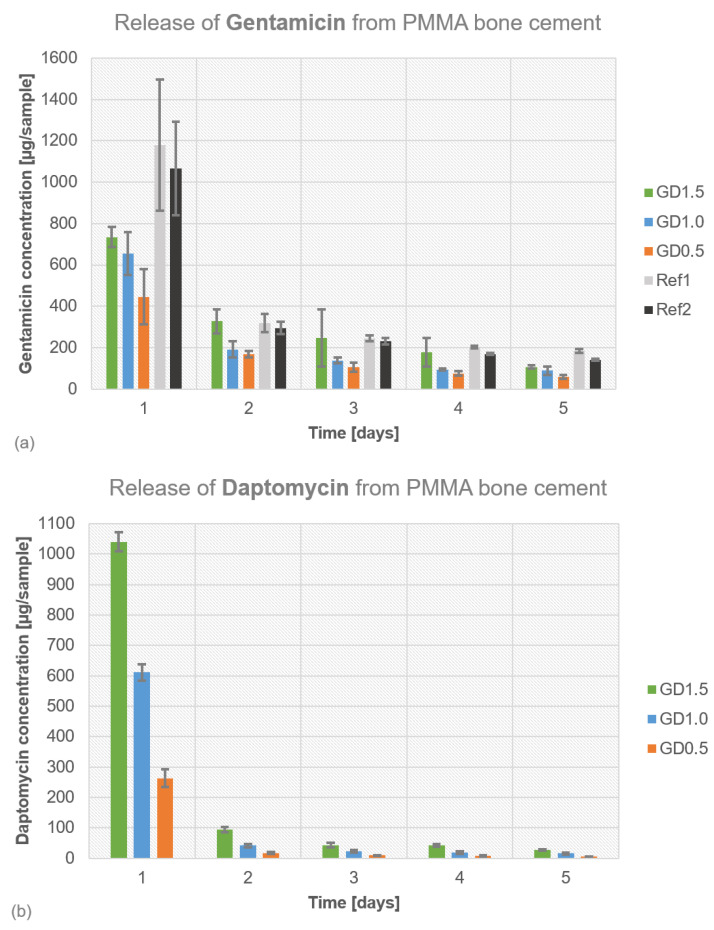

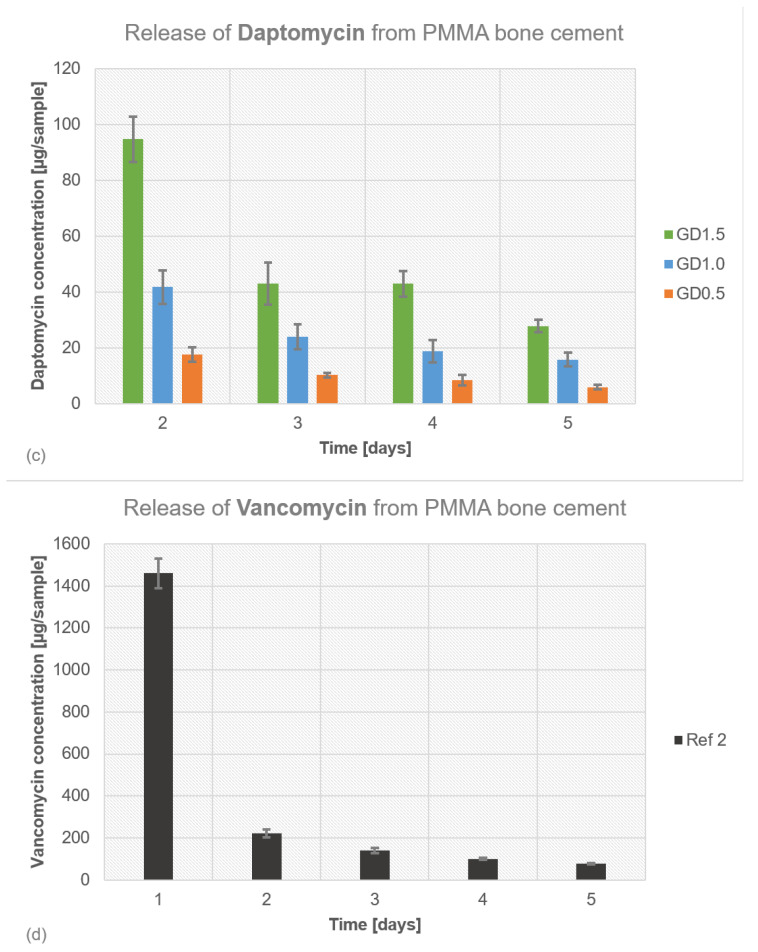
Release profiles from PMMA bone cement were determined with HPLC for (**a**) gentamicin days 1–5, (**b**) daptomycin days 1–5, (**c**) daptomycin days 2–5, and (**d**) vancomycin days 1–5. Ref1 (PALACOS^®^ R+G), Ref2 (COPAL^®^ G+V).

The highest volume of gentamicin released from daptomycin + gentamicin-loaded PMMA cement samples was observed on day 1, followed by a continuous decrease in antibiotic release (Figure 4a). The gentamicin release was higher for those samples with a higher daptomycin concentration: GD1.5 showed the highest gentamicin release over five days compared to GD1.0 and GD0.5, indicating a synergistic elution effect. PALACOS^®^ R+G and COPAL^®^ G+V showed the highest antibiotic release on day 1 compared to the test samples with manually admixed daptomycin. The total release of daptomycin was higher (1039.7 ± 31 µg) compared to gentamicin (734.1 ± 48 µg) for GD1.5 (Figure 4b,c). Adding 0.5 g of daptomycin resulted in a twofold higher antibiotic release rate: GD1.5 with 1039.7 ± 31 µg, GD1.0 with 611.4 ± 27 µg, and GD0.5 with 263.8 ± 28 µg. Ref2 (COPAL^®^ G+V) was assessed for vancomycin elution (Figure 4d), showing the highest initial release of all tested samples on day 1 (1460.2 ± 70 µg), followed by a massive decrease to 221.4 ± 19 µg on day 2. Compared to the GD samples and Ref1 (PALACOS^®^ (Wehrheim, Germany) R+G), Ref2 (COPAL^®^ G+V) showed the highest total amount of antibiotics eluted. Compared to the vancomycin release (Figure 4d) from Ref2 (COPAL^®^ G+V), the total amount of daptomycin released was lower, indicating an overall better elution from Ref2 (COPAL^®^ G+V) compared to the GD samples.

### 2.5. Mechanical Stability of Daptomycin-Loaded Bone Cement

**Figure 5:**
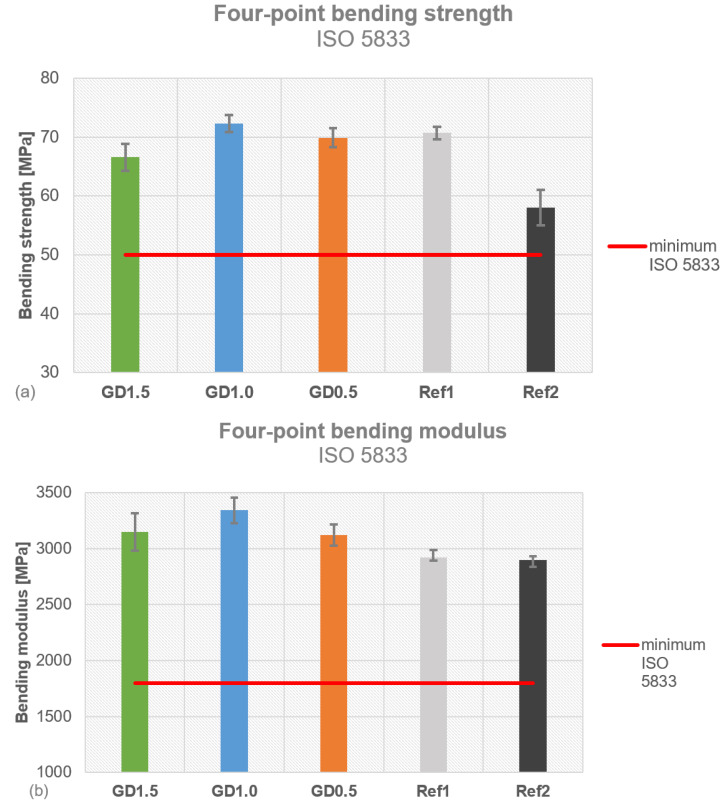

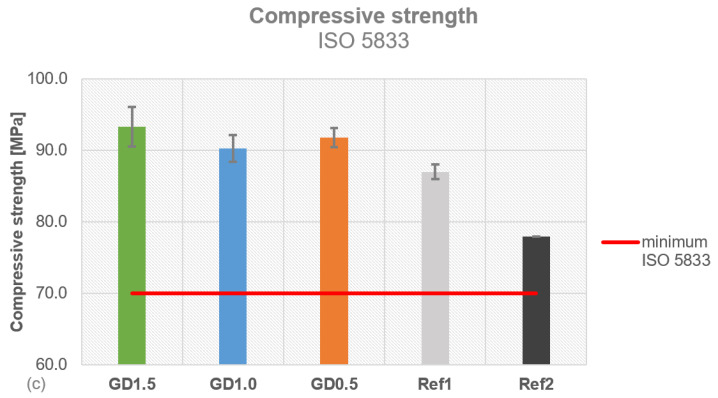
Mechanical stability tested for all daptomycin concentrations compared to PALACOS^®^ R+G and COPAL^®^ G+V: (**a**) four-point bending strength; (**b**) four-point bending modulus; (**c**) compressive strength.

All tested daptomycin-containing PMMA cement samples fulfilled the ISO and DIN requirements (Figure 5a). PALACOS^®^ R+G (Ref1) showed a bending strength of 71 ± 1 MPa, surpassing the threshold of 50 MPa. COPAL^®^ G+V (Ref2) was close to the threshold, with a bending strength of 58 ± 3 MPa. The ISO bending strength was highest for GD1.0 (72 ± 1 MPa), followed by GD0.5 (70 ± 2 MPa) and GD1.5 (67 ± 2 MPa), which therefore come with a higher bending strength than COPAL^®^ G+V (Ref2). The bending modulus of Ref1 (2922 ± 66 MPa) was comparable to that of Ref2 (2900 ± 30 MPa), fulfilling the minimum threshold of 1800 MPa (Figure 5b). GD1.0 had the highest bending modulus, with 3342 ± 113 MPa, followed by GD1.5 (3148 ± 168 MPa) and GD0.5 (3120 ± 95 MPa); all of the GD samples surpassed the references. Ref2 fulfilled the minimum requirement (70 MPa) for ISO compressive strength, with 78 ± 0 MPa, whereas Ref1 exceeded it (87 ± 1 MPa) (Figure 5c). The compressive strength was highest for GD1.5 (93 ± 3 MPa), followed by GD0.5 (92 ± 1 MPa) and GD1.0 (90 ± 2 MPa).

**Figure 6:**
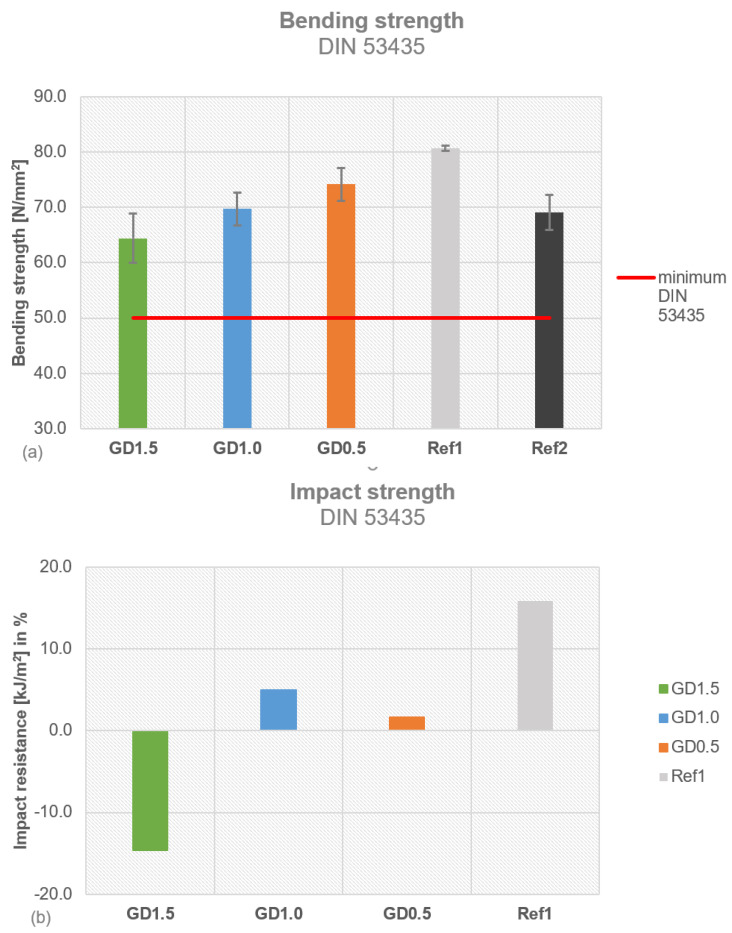
Mechanical stability tested for all daptomycin concentrations (GD1.5, GD1.0, and GD0.5) compared to PALACOS^®^ R+G (Ref1) and COPAL^®^ G+V (Ref2): (**a**) DIN bending strength; (**b**) DIN impact strength shown as the difference compared to Ref2.

All GD cement samples fulfilled the requirements for mechanical stability according to DIN 53435. Ref2 exceeded the minimum threshold (50 N/mm^2^) of DIN bending strength, with 69 ± 3 N/mm^2^, as did Ref1 with 81 ± 1 N/mm^2^ (Figure 6a). The DIN bending strength decreased with the increase in the daptomycin concentration, from 74 ± 3 N/mm^2^ (GD0.5) to 70 ± 3 N/mm^2^ (GD1.0) and 64 ± 4 N/mm^2^ (GD1.5). GD1.0 showed a comparable DIN bending strength to Ref2, whereas GD1.5’s was slightly below that of Ref2. The DIN impact resistance was measured for COPAL^®^ G+V (Ref2) as 3.0 ± 0.3 kJ/m^2^, which was set as a reference. The DIN impact resistance is shown as the difference compared to COPAL^®^ G+V (Ref2) (Figure 6b). Ref1 (3.5 ± 0.3 kJ/m^2^), GD1.0 (3.2 ± 0.4 kJ/m^2^), and GD0.5 (3.1 ± 0.5 kJ/m^2^) exceeded the impact resistance of Ref2, while GD1.5 (2.6 ± 0.6 kJ/m^2^) showed the highest difference in DIN impact resistance. The higher the daptomycin concentration, the lower the measurements for DIN bending strength and impact resistance, indicating that a high daptomycin concentration in PMMA bone cement reduces its mechanical properties. The ISO bending strength for GD1.5 was lower compared to all other concentrations and the references.

### 2.6. Handling Properties of Daptomycin-Loaded Bone Cement

GD1.5 was slightly faster-setting compared to COPAL^®^ G+V (Ref2), and even more so compared to PALACOS^®^ R+G (Ref1) (Table 1), resulting in a faster setting behavior. The ISO doughing time was lowest for Ref2 and highest for GD1.5, indicating a slower doughing process for GD1.5. The density of all tested cement samples was similar.

## 3. Discussion

As PJIs still pose a threat to patients and the healthcare system, especially when caused by resistant germs, all kinds of support are needed to prevent PJIs or treat them in a two-stage revision protocol [17]. Daptomycin-containing PMMA cement is recommended for PJIs when combatting vancomycin-resistant germs, e.g., VRE.

In clinical practice, spacers for two-stage revision procedures are created by using industrially manufactured cement already containing vancomycin, or by manually admixing vancomycin with PMMA cement [6,18,19]. The use of the commercially available cement COPAL^®^ G+V is already established for the prevention of PJIs, especially against the most frequent PJI germs *S. aureus* and *S. epidermidis* [8,18], but does not cover VRSA, VISA, or VRSE. In general, it’s recommended to use a fixation or spacer cement containing two complementary antibiotics to best cover the spectrum of PJI pathogens [12]. The spectrum of vancomycin is predominantly limited to Gram-positive bacteria; therefore, a broad-spectrum antibiotic is needed to also cover Gram-negative bacteria, e.g., gentamicin [13]. Additionally, the combination of two antibiotics offers a synergistic elution effect that leads to an overall increase in antibiotic elution from PMMA bone cement, resulting in a stronger antimicrobial effect [2]. Antibiotic-loaded bone cement with gentamicin and daptomycin can make a difference in the prevention and treatment of PJIs caused by vancomycin-resistant pathogens [20]. Daptomycin offers a good safety profile and has a unique mode of action that may also be effective against bacteria in biofilms [20], coming with a low resistance profile [21]. According to Gray and Wenzel [14], knowledge on the exact mode of action is still missing, but they observed that the development of resistance to daptomycin was slower compared to other antibiotics with single protein targets. Rouse et al. [22] figured out with a rat model that a PMMA cement with daptomycin may be an option for the local treatment of resistant bacteria causing osteomyelitis. The manual admixture of daptomycin with PMMA bone cement is suggested for PJI cases caused by VRSA, VRE, MRSA, and MRSE with a vancomycin MIC of greater than 2 µg/mL [10]. A first case study in 2013 already showed the ability of PMMA spacers with daptomycin to eradicate an infection in a two-stage revision hip surgery [23]. In our study, we found antimicrobial effectiveness for GD1.5 and GD1.0, whereas a concentration of 0.5 g of daptomycin was not sufficient. This is in line with findings from Eick et al. [24], who concluded from their inhibition zone testing that 1.5 g of daptomycin showed an antimicrobial effect, in contrast to 0.5 g. This may also be caused by the combination of two antibiotics (gentamicin and daptomycin) and the resulting synergistic elution effect. The combination of 0.5 g of gentamicin and 1.5 g of daptomycin showed the best antibiotic elution profile, as well as a synergistic elution effect supporting the prevention of infection [15]. The overall elution profile of daptomycin was comparable to the findings of Meeker et al. [25], showing peak elution for the first 24 h. Our observed synergistic elution effect contrasts with the results reported by Antonello et al. [20], who concluded a rather antagonistic interaction of daptomycin and gentamicin from their study review. But studies on a *Galleria melonella* larvae biofilm model also showed a synergistic effect of combining gentamicin with daptomycin for the treatment of vancomycin resistant *E. faecium* [26]. We observed an effect on the bacterial growth comparable to that reported by Webb et al. [27], indicating an inhibitory effect of daptomycin on the growth of resistant strains of Gram-positive bacteria. Overall, the synergistic elution effect and the high daptomycin release suggested a positive effect of combining 0.5 g of gentamycin and 1.5 g of daptomycin in PMMA bone cement. We also investigated the vancomycin elution, with COPAL^®^ G+V as a reference. Despite finding the highest vancomycin elution for day 1, the elution had already decreased by ~85% on day 2. We doubt that this vancomycin concentration, in a clinical setting, would be sufficient to meet the MIC of VRSA (MIC ≥ 16 µg/mL) and VRE (MIC ≥ 32 µg/mL) [10], but we would also suggest further investigations in a biofilm model.

To ensure patient safety, daptomycin must be sterilized with either ethylene oxide or gamma radiation. Our findings suggested that daptomycin-loaded bone cement should be sterilized using gamma radiation to maintain its antimicrobial effectiveness, because sterilization with ethylene oxide reduced the efficacy of daptomycin significantly.

To best treat MRSE, MRSA, or enterococci, the *PIF Pocket Guide* [16] recommends increasing the vancomycin concentration in commercially available COPAL^®^ G+V (40 g) by another 2 g. But an increase in the addition of antibiotic powder beyond a total concentration of 10% results in a spacer cement that no longer fulfills the mechanical ISO requirements for bone cement [15,19,28]. Despite Lunz et al. [29] pointing out that an antibiotic concentration exceeding 10% of the powder volume significantly reduces the mechanical strength of PMMA spacers, they recommend manually admixing 4 g of vancomycin with 40 g of PALACOS^®^ R+G instead of using the commercially available COPAL^®^ G+V. These spacers do not comply with the ISO requirements, as the bending strength for PALACOS^®^ R+G + 4 g of vancomycin is below the minimum threshold of 50 MPa and comes with the potential risk of bone cement or spacer fracture [15,19,21,29]. Therefore, we assessed the mechanical stability of a daptomycin-containing bone cement. The ISO bending modulus of the GD samples was increased compared to the reference samples, as admixing antibiotics increases the hydrophilic characteristics of PMMA cements, which results in increased elasticity of the cement [15]. The mechanical properties of GD1.5, GD1.0, and GD0.5 were all above the corresponding minimum thresholds, as the concentration of the added daptomycin was below 10% of the total PMMA cement powder volume [15,22,30]. Considering the mechanical properties, a concentration of 1.0 g of daptomycin would be ideal, but this does not offer a sufficient antibiotic release needed for preventing infection. As the bone cement GD1.5 showed antimicrobial effectiveness and promising mechanical properties, handling characteristics were only assessed for this bone cement sample. The ISO setting time, assessed according to ISO 5833:2002 [31], determines the timepoint when the bone cement is completely set and cannot be handled any longer. The ISO doughing time describes the time until the PMMA cement reaches the dough state. The ISO setting time and ISO doughing time were comparable to those of PALACOS^®^ R+G and COPAL^®^ R+V, indicating a slightly faster setting time for GD1.5, which means a shorter application time window.

**Figure 7:**
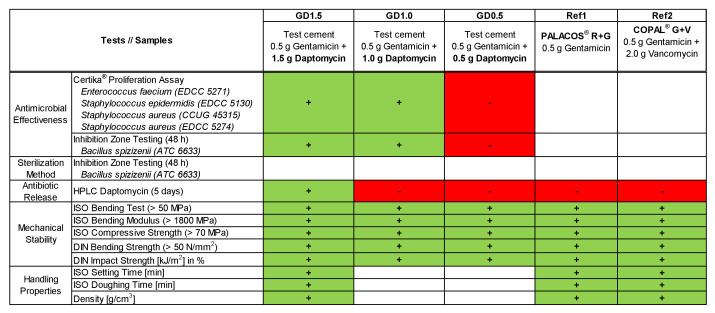
Overview of the tests performed to evaluate the best concentration of daptomycin that can be added to PMMA bone cement (color coding: white = not evaluated, green/+ = effective or requirements fulfilled; red/− = ineffective or requirements not fulfilled).

Our investigations indicated that the performance of a PMMA bone cement containing 0.5 g of gentamicin and 1.5 g of daptomycin is the optimal choice considering its antibiotic effectiveness, antibiotic release, and mechanical stability (Figure 7). According to the recommendations from the PRO-IMPLANT Foundation, 3.0 g of daptomycin can be added to a PMMA spacer cement made from 40 g of powder [16], but Kühn [15] suggested not adding more than 2 g of daptomycin, which is in line with our findings. PMMA spacers with manually added antibiotics must also fulfill the legal requirements for medical devices and comply with the ISO standards [15]. This is rather important from a legal perspective, as the surgeon becomes the legal manufacturer of the product by admixing antibiotics. We assumed that the concentration of 3 g of daptomycin was recommended because the antimicrobial effect was perceived as insufficient, so this indicates that the antibiotic release is too weak. It is also described in the literature that the “Daptomycin dose in ALBC for spacer should be 3.3-times the original dose to double the release” [21]. As the mode of action of daptomycin is dependent on calcium ions, solely adding more daptomycin does not necessarily improve the antimicrobial effectiveness [14]. The mode of action is dependent on a sufficient concentration of calcium ions [14] in the surrounding tissue; to increase the inhibitory effect of daptomycin eluted from PMMA bone cement, calcium ions could potentially be added [30]. We want to investigate this in a further study. As manually admixed ALBC is mainly used for spacers, we recommend investigating a longer period of more than 14 days to better simulate the spacer interim period of a two-stage revision protocol [19]. Despite this, we also recommend further investigations with daptomycin-containing PMMA cement in biofilm models.

We want to highlight that a surgeon ordering daptomycin from the pharmacy will receive a different product than the industrially used daptomycin. The clinical available “Cubicin” [32], in addition to daptomycin, also contains sodium hydroxide, the potential influence of which on the mechanical properties and antibiotic elution of PMMA bone cement is not yet clarified. Following the recommendations of the PRO-IMPLANT foundation [16], the addition of Cubicin is costly: for a fixation cement it costs ~780 EUR (2 g), and even more for a spacer cement (~1500 EUR (3 g)), with including the price for a gentamicin-loaded PMMA bone cement as basis for admixing. From a financial perspective, a commercially available PMMA bone cement with gentamicin and daptomycin could be of interest.

## 4. Materials and Methods

### 4.1. PMMA Cements and Bacteria

**Figure 8:**
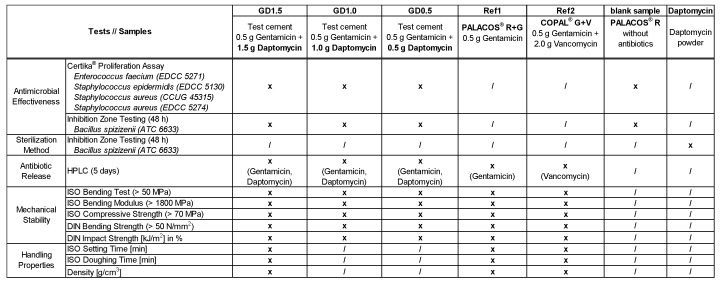
PMMA bone cement test samples and the tests performed to determine their antimicrobial effectiveness, antibiotic release, mechanical stability, and handling properties (x = tested; / = not tested).

PALACOS^®^ R, PALACOS^®^ R+G, and COPAL^®^ G+V (Heraeus Medical GmbH, Wehrheim, Germany) were used. PALACOS^®^ R is a plain PMMA cement without antibiotics, PALACOS^®^ R+G contains 0.5 g of gentamicin, and COPAL^®^ G+V contains 0.5 g of gentamicin combined with 2 g of vancomycin. PALACOS^®^ R+G was loaded with 1.5 g, 1.0 g, and 0.5 g of daptomycin powder (Xellia Pharmaceuticals ApS, Copenhagen, Denmark). PALACOS^®^ R, PALACOS^®^ R+G, and COPAL^®^ G+V were used as references (Table 1). Daptomycin was manually admixed at three different concentrations of 1.5 g (GD1.5), 1.0 g (GD1.0), and 0.5 g (GD0.5). Test strains derived from clinical isolates from the Eugen Domann Culture Collection (EDCC) and Culture Collection University of Gothenburg (CCUG), with different resistance patterns against gentamicin, methicillin, and vancomycin, were used to test the antimicrobial properties of the bone cement samples in vitro (Figure 8).

### 4.2. Certika^®^ Proliferation Assay

The Certika^®^ microplate proliferation assay was used to determine the antimicrobial efficacy of material surfaces by measuring their ability to prevent the multiplication of microorganisms and germs on a surface. The testing was conducted according to a method developed by QualityLabs BT GmbH, published by Bechert et al. in 2000 [33,34,35]. Bone cement samples (seven replicates each) with a diameter of 6 mm were prepared using molds. The tested material was defined as antimicrobial if the formation of at least 99.9% of the daughter cells during the observation time was prevented in comparison to the blank sample. Statistical analysis was performed in line with DIN EN ISO/EC 17025 [36].

### 4.3. Inhibition Zone Testing

To detect the antimicrobial effectiveness, inhibition zone tests were performed. Agar plates with Bacto agar (2% agarose) and Tris-buffered minimum medium (Ca 10 µM, phosphate 130 µM) were prepared and incubated with *Bacillus subtilis* ssp. *Bacillus spizizenzii* ATCC 6633 [37]. Two bone cement samples with a diameter of 6.0 mm were placed on one plate, executing four repetitions per cement sample. The agar plates were incubated for 48 h at 36 °C. IMAGE pro scanning software was used to determine the sizes of the inhibition zones, as well as for statistical analysis. Afterwards, the average values and standard deviations were calculated for all of the test samples. If daptomycin was effective against *B. subtilis*, it would diffuse in the agar, creating a clear area, known as the zone of inhibition. In this clear zone, the growth of the bacteria is inhibited. The size of this zone was measured and used to interpret the effectiveness of PMMA bone cement containing daptomycin as an antimicrobial agent.

To determine the influence of sterilization on the antimicrobial effectiveness of non-sterilized, ethylene-oxide-sterilized, and gamma-sterilized daptomycin against *B. subtilis*, inhibition zone tests were performed by an external certified lab (INNOVENT e.V. Jena). Data collection and statistical analysis were performed using IMPAGE pro V2 scanning software.

### 4.4. High-Performance Liquid Chromatography (HPLC)

To determine the release profile of gentamicin and daptomycin dissolved from bone cement samples over 5 days (60 dissolution samples each), HPLC with MS/MS detection was performed [38,39]. As the medium for dissolution, 0.1 M Tris-hydrochloride buffer (pH 7.4) was used. Gentamicin was determined by analyzing its major components separately (resulting in three signals/peaks during LC-MSMS) and summing these three signals to a total concentration. Daptomycin was directly determined using LC-MSMS. The method was validated online using one set of matrix calibration standards and two sets of quality control samples. The calibration samples were used to calculate the results, and the quality control samples were used to monitor the quality of the analytical run. The mean values and standard deviations were calculated for all of the test samples.

### 4.5. Mechanical Stability Testing According to ISO 5833 and DIN 53435

To ensure that the added daptomycin did not negatively influence the mechanical stability of the PMMA cement, mechanical tests for bending strength, bending modulus, and compressive strength were performed according to ISO 5833:2002 [31]. To determine the compressive strength, the cement rods (12 mm height, 6 mm diameter) were loaded with a constant crosshead speed of 19.8–25.4 mm/min. The tests were run at 23 ± 1 °C with dry specimens prepared 24 h before testing. For the bending strength and bending modulus, rectangular specimens (3.3 mm × 75.0 mm × 10.0 mm) were used; they were loaded with a constant crosshead speed of 5 mm/min. The tests were run at 23 ± 1 °C with dry specimens prepared 24 h before testing. The four-point bending test rig had 60 mm between the outer loading points and 20 mm between the inner loading points. The tests were continued until failure, and the maximum force was used to calculate the bending strength. Value calculations and statistical analysis were performed as described in the ISO standard [31].

The DIN impact strength and bending strength were also determined according to DIN 53435 [40]. The rectangular specimens (3.0 mm × 15.0 mm × 10.0 mm) were stored for at least 12 h under standard climatic conditions using the appropriate impact direction (i.e., consumption of at least 10%, and at most 80%, of the maximum impact by the test specimens). The bone cement samples were placed vertically in the test device, and the pendulum was adjusted to 90° and the height of the drop. According to DIN 53435 [40], the average and standard deviation were calculated in kJ/m^2^. For the DIN bending strength, a bending force of 400 Ncm was applied to the bone cement samples until they broke. The bending strength was measured and calculated, and statistical analysis was performed according to DIN 53435 [40].

### 4.6. Handling Properties of PMMA Cement

The ISO setting time and ISO doughing time were assessed, and statistical analysis was performed [31].

## 5. Conclusions

Our results suggest adding 1.5 g of daptomycin to PMMA cement combined with 0.5 g of gentamicin for PJI cases caused by vancomycin-resistant germs. All mechanical and handling properties, along with the elution profile and effectivity of 1.5 g of daptomycin added to 40 g of PMMA, fulfilled all clinical requirements. Due to its antimicrobial spectrum against vancomycin-resistant germs (e.g., VRE), a PMMA cement containing 0.5 g of gentamicin and 1.5 g of daptomycin instead of vancomycin could be a good option for the treatment of PJIs. Further investigations of its performance in biofilm models and against clinical isolates are recommended.

## Figures and Tables

**Table 1:** Handling properties of PMMA bone cement containing 0.5 g of gentamicin + 1.5 g of daptomycin compared to commercially available bone cements Ref1 and Ref2.

Handling Properties	GD1.5	PALACOS^®^ R+G Ref1	COPAL^®^ G+V Ref2
ISO Setting Time (min:s)	06:45 ± 00:00	09:30 ± 00:10	08:15 ± 00:18
ISO Doughing Time (min:s)	01:30 ± 00:00	00:55 ± 00:05	01:00 ± 00:05
Density (g/cm^3^)	1.13 ± 0.01	1.15 ± 0.00	1.12 ± 0.01

## Data Availability

All data are presented in this article. The data are also available from Heraeus Medical GmbH.
